# Supplementary material for: Are carbon emissions trading and green financial instruments synergistic? -Comprehensive quantitative research based on content analysis
Source: PLoS One. 2024 Mar 7;19(3):e0298601. doi: 10.1371/journal.pone.0298601 (PMC10919601; doi:10.1371/journal.pone.0298601)
Supplement: S1 Data — (ZIP) [file pone.0298601.s001.zip › Data/地级市Z_F工作报告.zip!/┴╔─■/┼╠╜⌡╩╨/└┤╫╘╣1⁄2╓┌║┼í╛┬φ┐╦╩2╛▌═°í┐_2014.docx]

各位代表：

现在，我代表市人民政府，向大会报告工作，请予审议，并请市政协委员提出意见。

一、过去四年工作回顾

市六届人大一次会议以来的四年，是极不平凡的四年。在市委的正确领导下，我们紧紧依靠全市人民，开拓创新，真抓实干，经受住 了前所未有的困难和考验，经济社会发展取得重大成就，在盘锦发展史上书写了新的辉煌！

(一）经济总量实现新跨越

2013年，全市地区生产总值完成1360亿元，是2009年的2倍，年均增长13.1%（按不变价计算）。公共财政预算收入148.8亿元，是2009 年的2.8倍，年均增长29%。固定资产投资1140亿元，是2009年的2.8倍，年均增长29%。社会消费品零售总额286亿元，是2009年的 1.8倍，年均增长16.2%。外贸出口8亿美元，是2009年的2.7倍，年均增长28.7%。实际利用外资15亿美元，累计完成60.2亿美元，总量 连续四年排名全省第三。

四年来，各项主要经济指标持续走在全省前列，综合实力显著增强，人民生活幸福指数大幅提升，盘锦发展实现了历史性突破！

(二）区域发展开创新局面

——坚持向海发展、全面转型、以港强市，辽东湾新区在盐碱荒滩上拔地而起，基础设施、公共设施不断完善，长春石化、宝来石化、和运橡胶、渤海装备、合力叉车等项目建成投产，实现港城融合、联动发展。新区已成为辽宁沿海发展最快的重点园区，已晋升为国家级开发区。

——港口建设步伐加快，30万吨原油码头建设实现突破，建成10个5万吨泊位，港口吞吐量达到3000万吨。铁路、公路等集疏运体系配套形成，盘锦港成为东北及蒙东地区最近的出海通道。

——5个生态新城全面推进，5个重点工业园区快速发展，辽河口生态经济区、红海滩旅游度假区加快建设，高升经济区成为国家级现代农业示范区。

——县域经济成为新的经济增长点，城区经济迸发出新的活力，两县两区在全省位次大幅前移。

今天的盘锦，以辽东湾为龙头的发展格局已经形成，县区和经济区竞相崛起，一座充满自信、充满希望的港口城市正大步走向世界，走向未来。

(三）用项目推动三次产业跃上新台阶

累计新上亿元以上项目410个，建成投产293个，其中10亿元以上项目124个，产业转型升级取得重大进展。

1.新型工业化进程加快。规模以上工业增加值年均增长15.6%。引进世界500强、行业100强企业19户，40万吨润滑油、18万吨醋酸仲丁酯、16 万吨EPS、15万吨丁二醇等一批大项目建成投产，新增百亿级企业３户，建成2个国家级新型工业化示范基地，石化及精细化工产业集群在全省率先突破千亿规 模，装备制造产业集群实现倍增，建设世界级石化及精细化工产业基地、国内领先的能源装备制造基地迈出重要步伐。辽河油田、华锦集团、辽河石化等大企业为盘 锦发展做出重大贡献。以科技引领产业发展，CP系列钻井平台、卤化丁基橡胶等一批高新技术实现产业化，高新技术产品增加值年均增长20%。

2.现代服务业蓬勃发展。以旅游业拉动服务业发展，形成7个省级服务业集聚区，兴隆台中央商务区成为省级服务业示范区，“金廊银带”成为辽宁旗舰型服务业 集聚区。温泉产业蓬勃兴起，红海滩风景廊道展现无穷魅力，旅游总收入年均增长36%。鹏欣水游城、大商商业综合体等投入运营。红星国际建材博览中心、中国 食品城、蓝碧源五金汽配城建成使用，专业市场建筑面积超过200万平方米。成功举办湿地旅游周、装备博览会、稻米交易会等大型会展活动，城市知名度空前提 升。

3.农业现代化步伐加快。粮食生产连续10年获得大丰收。益海嘉里、天津宝迪、江西正邦、浙江森禾等一批龙头企业进入盘锦，农业产业化大项目建设走在全省 前列。水稻生产综合机械化率和土地流转率均居全省首位。河蟹产量保持全国地级市之首。大米河蟹品牌价值大幅提升，北京展销活动取得巨大成功，盘锦已成为中 国北方最大的河蟹销售集散地，成为东北稻米交易中心之一。

(四）城乡面貌发生新变化

1.大城市框架全面拉开。高标准编制城市总体规划，5个生态新城建设全面展开、各具特色，香港东方银座、江苏苏宁、广东美的、珠海华发、北京泛华、广东富 盈、上海绿地等国内外知名的大企业大集团成为城市建设的主力，建成区由89平方公里扩大到154平方公里。新市镇建设步伐加快，盘锦进入城乡一体化发展新 阶段。

2.基础设施建设取得重大进展。盘营客专开通运营，阜盘高速建成通车，向海大道、中华路、305国道成为盘锦新的发展动脉。盘锦新站建成使用，辽东湾互通 立交、环城路、惠宾大街等56项重点交通工程相继竣工，新建、改造道路里程超过1800公里。大伙房水库输水配套工程全线供水，华润城市热电一期工程如期 推进，电网改造、城市管网等工程加快建设，基础设施支撑发展的能力显著增强。

3.生态环境质量全面提升。资源保护成效显著，盘锦湿地成为“中国十大魅力湿地”之一。新增造林绿化面积15.2万亩，新建城市公园15座，全市林木绿化 率提高到10.9%，建成区绿化覆盖率达到41.1%。辽河流域治理成效显著，退耕还河5.9万亩，建成乡镇污水处理厂17座，辽河干支流水质全部达到四 类标准。大力推进蓝天工程，空气环境质量综合指数排名全省前列。节能减排实现控制目标。全国文明城市创建工作取得重要的阶段性成果。盘锦向着国家级生态园 林城市的目标大步迈进。

(五）改革创新迈出新步伐

——完成行政区划调整，拓展了城市发展空间，组建辽东湾新区和辽河口生态经济区。

——大力简政放权，累计向县区和经济区下放市级行政权限504项，盘锦成为全国权力下放最为彻底的地级城市之一。

——成立6个特色园区，建立了全新的选人用人机制和管理模式。

——创新招商机制，组建市县（区）两级专业招商机构，形成系统化、专业化的招商体系。

——创新融资机制，构建多元化融资体系，全市年均新增融资总量200亿元以上。

——创新土地管理机制，土地供应有力支撑经济社会发展，盘锦成为全国土地管理创新试点市。

体制机制创新释放了能量、激发了活力，在转型发展的实践中，广大干部群众始终激情满怀，干劲十足。

(六）人民生活水平有了新提高

坚持民生优先，全市财政用于民生支出累计达到431亿元，年均增长29.4%。

——居民收入：农民人均纯收入达到14617元，是2009年的1.7倍。城镇居民人均可支配收入达到30562元，是2009年的1.6倍。

——居民就业：累计实名制就业27.3万人次，城镇登记失业率保持在3%以下，始终好于全省平均水平。零就业家庭保持动态为零。

——社会保障：实现基本养老和医疗保险制度城乡全覆盖，城乡居民和城镇职工社会保险待遇逐年提高。社会救助体系不断健全，在全省率先实施城乡一体的低保救助，县域低保标准提高1.5倍。

——教育事业：义务教育均衡发展取得重要进展，职业教育、高中教育、特殊教育、学前教育有了长足进步。大连理工盘锦校区开学运行，开启了盘锦高等教育发展的崭新篇章。

——文化体育：文化事业和文化产业繁荣发展，红海滩入选《美丽中国》系列邮票，市民文化生活日益丰富。红海滩体育中心成功承办“十二运”竞赛项目，全民健身中心、老干部活动中心落成使用，群众性文化体育活动蓬勃开展。

——医药卫生：城乡医疗卫生服务体系逐步完善，国家基本药物制度惠及全市人民，在全省率先启动大病保险制度和新农合“一卡通”。高标准建成市中心医院、盛京医院盘锦分院。

——安居惠民：落实保障性住房10.6万套。解决37万农村居民饮水安全问题。新改建街路174条，修缮农村公路631公里。农村校车配备、市民房屋产权办理、公交车辆更新、老旧小区维修改造等一系列民生问题得到有效解决。全市人民不断增进福祉、共享发展成果。

(七）民主法制和政府自身建设取得新进步

自觉接受人大及其常委会的法律监督、工作监督和政协的民主监督，虚心听取各方面意见和建议，认真办理人大代表议案、建议和政协提案。

全面推进依法行政。坚持依法科学民主决策，强化政府投资项目、财政资金、公共资源交易等制度管理，不断加大政务公开、行政监察、审计监督力度。深入开展纠 风工作，积极推动惩治和预防腐败体系建设。持续推进机关效能建设，行政效率和服务水平显著提高。盘锦连续四年排名全省绩效考评前列。

创新社会治理服务，狠抓社会稳定、安全生产和食品药品安全工作，加强政府应急管理，完善社会治安防控体系，社会矛盾得到有效化解，平安创建活动扎实推进，盘锦荣获“长安杯”。

国防教育、国防动员和双拥共建深入开展，军政军民团结的局面进一步巩固。人口计生、妇女儿童、广播电视、老龄慈善、扶贫助残等事业不断发展，民族宗教、外事侨务、防震减灾、气象人防、统计、物价等工作取得了新的进步。

各位代表：回顾四年历程，我们倍感鼓舞！全市上下风雨同舟、砥砺同行，以令人瞩目的业绩，成就了一个辉煌的四年。所有这些成绩的取得，归功于省委、省政府 和市委的正确领导，归功于全市广大干部群众和人大代表、政协委员的团结奋斗，归功于各民主党派、工商联、人民团体和社会各界的广泛参与，归功于驻盘各单 位、人民解放军、武警部队的大力支持。在此，我代表市人民政府，向所有关心、支持和参与盘锦改革发展的同志们、朋友们，表示衷心的感谢和崇高的敬意！

在总结工作的时候，我们也清醒地看到，经济社会发展和政府工作还面临许多困难和问题，主要是：经济发展潜力还没有得到充分释放，发展不平衡问题突出；结构 调整需要进一步优化，县区工业产业集群规模不大，生产性服务业发展滞后，民营经济发展还不够充分，外贸依存度偏低，自主创新能力不强；社会治理与公共服务 水平亟待提高，改善民生任务繁重；软环境建设有较大差距，行政服务效率需要进一步提高，政府自身建设有待加强。对这些问题，我们一定认真加以解决。

二、全面推进深化改革

各位代表，新一届政府任期的四年，是全面深化改革的关键期，是盘锦实现宏伟蓝图的决胜期。今后四年工作的总体要求是：深入贯彻落实党的十八大、十八届三中 全会精神，按照市委确定的宏伟蓝图，全面深化改革，不断扩大开放，促进社会公平正义，切实提高经济发展质量和效益，加快向海发展全面转型以港强市步伐，推 动经济社会发展持续走在全省前列，建设世界级石化及精细化工产业基地、具有吸引力的国际旅游胜地和世界知名的港口城市，建成让全市人民普遍感到幸福、引以 自豪的滨海新盘锦。

实现奋斗目标，关键靠改革。我们要从现在做起，从能做的事情做起，勇于探索创新。

1.推进各类市场体系建设。建立城乡统一的建设用地、人才、技术等市场，组建政府服务采购、工程建设招投标管理中心，发挥市场的决定性作用。推进社会诚信体系建设。

2.推进市场准入制度改革。取消企业注册等限制条件，降低创业门槛，促进民营经济大发展。完善项目备案，取消企业投资一般性项目审核制度。先行先试放宽外资投资准入。

3.推进国有企业和公用事业单位改革。完善国有资产管理体制，组建国有资本运营投资公司。完善价格形成机制，规范土地价格。深化水价、燃气价格改革，逐步实行阶梯价格。

4.推进政府职能转变。整合优化政府内部机构设置，实现一项工作一个部门负责。加快事业单位分类改革。完成政府部门与中介机构脱钩分离，实现中介机构市场化。

5.推进行政审批制度改革。推行部门权力清单制度，明晰行政层级权责边界，做到该向市场和社会放开的领域全部放开，同时强化下放权力的规范运行。

6.推进财政体制改革。建设公共财政体系，完善预算管理制度。规范专项资金使用和管理。建立政府债务管控机制，防范和化解债务风险。

7.推进金融体制改革。发展债券、产权交易等多层次资本市场。以利民便民惠民为宗旨，建立多样性、多元化、多层次的金融服务体系。打造信用金融城市。

8.推进对外开放。加强软环境和硬环境建设，建设辽宁乃至全国最优的发展区和创业区。创新招商体制机制，建设一支有水平、懂业务的招商队伍。鼓励支持企业走出去，形成外贸出口的激励机制。

9.推进经济区和园区体制创新。围绕软环境建设，大力推进经济区、各类园区体制机制创新。全力争取国家批准设立辽东湾综合保税区，纳入国家自由贸易区。

10.推进科技体制改革。创新科技资金配置模式，集中力量支持重大技术攻关。加快建立产学研协同创新机制。加快形成以激励创新为导向的人才制度、专业技术职称评价制度和薪酬分配制度。

11.推进生态文明制度建设。完善环境治理和生态修复制度，加强湿地、海洋、河流保护，实施更加严格的监管制度。建立乡村环境管理体制和运行机制。

12.推进城乡要素平等交换和公共资源均衡配置。促进农村产权和土地承包经营权流转，均衡配置教育、文化、医疗等公共服务资源，建立全社会房产、信用等基础数据统一平台，鼓励社会资本参与城乡公用设施投资运营，扎实开展城乡一体化综合改革试验区建设。

13.推进户籍制度改革。放开户籍管理限制，改革社会保障体制和相关制度，让进城落户农民享有市民待遇。

14.改进绩效考核制度。除了继续考核主要经济指标外，要加大资源消耗、生态环境、科技创新、安全生产、政府性债务等指标权重。

15.强化权力运行制约监督。依法公开权力运行流程，完善惩治和预防腐败体系。完善作风建设常态化制度，以优良的政风行风带动社风民风。

各位代表：改革是渐进的过程，只有进行时没有完成时。我们要以强烈的进取意识、责任意识和法律意识，依靠全市人民，不断把改革引向深入。

三、2014年工作安排

各位代表：今年是盘锦建市30周年，是新一届政府的开局之年。我们要全面贯彻落实市委六届六次全会精神，坚持稳中求优，抓住六个关键，不断促进经济持续健康发展、社会和谐稳定。

抓发展：发展仍然是解决盘锦所有问题的关键。牢牢抓住发展这个第一要务，着力提升发展质量和效益，实现可持续发展。

抓改革：把改革贯穿于各个领域各个环节，把握全局，稳步推进，始终让盘锦充满活力。

抓项目：抓项目就是抓开放，抓发展，抓民生。坚持力度不减，抓项目、抓招商引资。

抓生态：生态环境关系人民福祉和盘锦未来。下决心抓好生态环境建设和保护，在发展中让全市人民享受碧水蓝天。

抓民生：始终把民生放在心上、抓在手中，全力为群众办实事、办好事，让全市人民的生活过得越来越好。

抓落实：抓而不实，等于白抓。把抓落实放在工作第一位，求实、务实、落实。

今年的主要预期目标是：地区生产总值增长9%；公共财政预算收入增长6%；固定资产投资增长16%；社会消费品零售总额增长13.5%；实际利用外资8亿 美元；外贸出口8亿美元；城镇居民人均可支配收入增长9%；农民人均纯收入增长10%。在实际工作中，力争各项主要经济指标增速走在全省前列。

(一）加快辽东湾新区建设

全面提升新区的龙头作用，各项工作走在辽宁沿海重点园区前列。

1.项目建设和招商引资实现新突破。全力支持兵器“十二五”项目开工建设，推进长春石化、和运橡胶、合力叉车等项目达产增效，加快北燃新厂、联成化学等项目建设。以更大力度和气魄抓招商，引进一批精细化工、装备制造、高新技术、临港物流和服务业大项目。

2.港口发展实现新突破。确保30万吨原油码头、25万吨航道工程取得突破性进展，新建、续建10个5万吨以上泊位。建成疏港高速，抓紧推进沈盘铁路，加快内陆港和集装箱航线建设，提升港口集疏运和服务功能，港口吞吐量力争达到4000万吨。

3.新城建设实现新突破。以规划引领新城建设，打造具有欧陆风情特色的低密度生态水城。完善城市基础设施和公共服务设施。用大企业大集团推进新城开发，加快瑞唐世贸中心、华源时代城、二界沟渔村改造等项目建设，集聚商气人气，形成新的商业中心和旅游热点。

4.体制机制创新实现新突破。创新园区管理机制，建立以业绩为核心的绩效考评体系，做到干好干坏不一样。创新审批服务机制，更加高效地为企业服务，建设最具活力、最有效率的国家级开发区。

5.要素保障实现新突破。加大资本运作力度。加强土地管理，抓好未利用地，提高土地产出率。支持大连理工盘锦校区、市职业技术学院建设，培育壮大人才队伍。

(二）全力推进工业发展

把精细化工和能源装备作为主攻方向，全面推进“五项工程”，规模以上工业增加值增长9.5%。

1.狠抓两大基地建设。

——加快建设世界级石化及精细化工产业基地，支持40万吨润滑油、18万吨醋酸仲丁酯、17万吨C5C9深加工等项目达产达效，推进戴纳索丁苯橡胶、胜友顺丁橡胶、飞扬化工等项目取得重大进展，发展高端润滑油、精细化学品、合成橡胶、合成树脂和工程塑料产业。

——加快建设国内领先的能源装备制造产业基地，支持渤海装备、忠旺铝材、奥德燃气等项目加快建设，引进特种压裂车、深水钻井平台等一批能源装备项目，发展石油天然气装备、海洋工程装备、临港装备产业。

——深化油地融合，全力支持辽河油田打造“双千”油田。促进辽河石化、长城钻探等企业做大做强，鼓励多种经营企业改革发展壮大。

——推动华锦集团、北方沥青等企业加速扩张，以龙头企业吸引项目集聚，向产业链要效益、要竞争力。

2.大力培育县区工业产业集群。工业产业集群是县区发展的支撑。突出专业化、集群化，推动精细化工、石油装备、新材料等产业集群加快发展，培育一批龙头企 业。大力发展工业地产。加强园区供水、供电等配套设施建设，搭建研发、检测、物流等公共服务平台，提高项目的承载能力。

3.加快科技创新。推动高新技术产业化和产业技术进步，支持企业开发新产品，培育一批小巨人企业，高新技术产品增加值增长20%以上。推进大连理工国家实 验室建设，支持陆海研究院、和运橡胶研究院等企业研发机构发展，支持企业海外并购。加快高新技术产业园建设，抓好曙光计算机产业基地等项目，培育发展新兴 产业。

(三）大力发展现代服务业

服务业是盘锦的“短板”也是最大的发展潜力，以旅游业为龙头，加快服务业发展。

1.加快建设具有吸引力的国际旅游胜地。规划、整合旅游资源，加快红海滩、辽河口度假区和鼎翔风景区建设。建成恒隆温泉、天沐温泉，加快富盈、海泉湾、海 世界等温泉旅游项目建设。开业铂尔曼酒店，推进万豪、君悦等酒店项目取得实质性进展，支持度假型酒店发展。推进旅游与文化融合发展，加强地域文化宣传，办 好湿地旅游周、河蟹文化节、冬捕节等活动，推出精品线路，拉长旅游周期和消费链条，千方百计留住游客。

2.加快中央商务区及服务业集聚区建设。以兴隆台中央商务区为龙头，突出抓好大连万达城市广场、武汉华氏中华城、左岸文化城、台北士林不夜城、金宝度假城 等重大项目。发展夜经济、楼宇经济，推进社区商业、便民商业，每个县区都要规划建设一个有特色的服务业集聚区，发挥聚集效应、拉动效应。

3.加快商品市场建设。以大洼商品市场集聚区为龙头，全面推进商品市场建设，完善功能配套，重点抓好红星国际建材博览中心、中国食品城、义乌国际商贸城、博景自由港等重大项目，形成辐射东北的商品交易集散中心。

4.保持房地产业健康发展。调整优化房地产结构，加快发展商业、旅游、温泉、养老、教育等可持续地产项目，高度重视规划、质量、环境和物业管理。继续搞好 房交会等促销活动。加大市场开拓力度，吸引外地人到盘锦购房、消费。房地产投资增长13%，商品房销售额增长11%。

5.大力发展现代物流业。推进益海嘉里、北京首农、双益公路港等物流项目加快建设，突出抓好辽东湾和双台子两个省级物流示范园区。鼓励支持辽河油田、华锦 集团、长城钻探等大型工业企业主辅分离，精心办好装备博览会、食品博览会等大型展会，积极发展电子商务，做大做强生产性服务业。

(四）加快发展现代农业

以产业化经营为重点，推进农业对外开放，加大农业投入，促进农业增效、农民增收。

1.着力推进农业产业化。加速高升国家级现代农业示范区建设。突出抓好辽宁田园、汇福粮油、香港宏安等10亿元以上农业产业化项目。发展新型农业经营主 体，家庭农场达到260个以上，农民合作社突破1000家，组建农业专业化服务公司。推进土地规模化经营，进一步提高土地流转率。

2.着力发展生态高效农业。不断提升耕地质量，稳定提高粮食产量。加快发展设施蔬菜，全力支持苇田养殖，抓好每日渔业、碱地蔬菜等项目建设，培育一批生态 农业品牌。加强绿色生产，强化疫病防治，从源头上保证农产品质量安全。扩大农业保险规模和险种。加强农田水利基础设施建设，提高农业综合开发水平，用科技 推动农业发展。

3.进一步叫响盘锦大米、河蟹两大品牌。走精品路线，推进水稻生产标准化、生态化、机械化，发展大蟹养殖，建设中国北方稻米交易中心和全国河蟹销售集散地。拓展大米河蟹销售渠道。继续办好北京大米河蟹展销会等活动，让盘锦大米河蟹走向全国、走向世界。

(五）建设生态文明宜居城市

坚持生态统领城乡一体化发展，建设美丽盘锦。

1.稳步推进新型城镇化。坚持规划先行，突出生态特色，构建现代、文明、和谐的城市格局。注重保留城镇风貌，形成一批特色新城、特色镇。以工业化、农业现代化推动城镇化，集中力量建设5个生态新城。加快农业转移人口市民化，推进以人为核心的新型城镇化建设。

2.全面推进棚户区改造。坚持市场主导、政府引导，抓住国家政策支持机遇，用有实力、有信誉的大企业参与改造，尽快完成全市棚户区和城中村改造任务，全面提升城市建设水平，让棚户区改造成为拉动发展的重大工程，成为改善民生的重大工程，建设辽宁棚户区改造示范市。

3.完善基础设施建设。完成地下管网普查。切实抓好盘锦北互通立交、辽河堤顶路、林丰路大桥等交通工程，加快改造农村路网和城区街路。推进大伙房输水配套 工程向乡镇延伸。启动华润城市热电二期，加快辽东湾热电、鹤乡500千伏输变电等工程建设。启动辽河盘山闸改造。进一步完善供水、供气、供热、供电等城市 功能配套，同时加强基础设施管护。

4.强化生态建设。编制生态规划，严守生态红线。扩大绿色空间，建设城市水系，创建国家级生态园林城市。加强芦苇湿地水利设施建设，保护好、利用好湿地资 源。狠抓蓝天工程，加快气化盘锦，抓好集中供热、大气监测等整治措施。继续加强辽河流域治理，运行乡镇污水处理厂，加快建设第三污水处理厂。搞好农村环境 治理。扎实有效推进造林绿化。完成节能减排任务。强化环境监管，从严查处环境违法行为。

5.加强城市管理。进一步加大全国文明城市创建力度，推进数字城市建设，形成精细化、常态化管理机制，全面提高城市管理水平和市民文明素质，展示盘锦的城市风采。

(六）坚定不移推进项目建设

抓项目，是盘锦发展的关键。确保项目引进、项目开工和项目竣工实现新突破。

1.全面提升招商质量。更加注重招商选资，主动对接世界500强、行业100强和国内外上市公司，实行点对点招商，全力引进一批调结构、促转型、增后劲的重大项目和高新技术项目。

2.狠抓项目开工。建立市县（区）两级责任推进机制，以项目开工为核心，全面做好审批服务和项目配套，更加主动地帮助企业解决问题，确保项目快开工、快建设、快竣工，早日达产达效。

3.全力支持现有企业发展。现有企业是现实的生产力。全力支持各类企业开展合资合作、并购重组、转型升级，扶持中小企业加快发展，加速壮大民营经济。

4.全力做好要素保障。创新融资方式，多渠道筹措资金。做大做强市商业银行，引进金融机构6家，切实帮助中小企业解决“融资难”问题。抓好国家土地管理创 新试点工作，强化土地节约集约，最大程度发挥土地使用效益。把集聚人才人口作为一项战略工程，加大金融、经济管理、专业技术等人才引进和培养力度，加速人 才人口向我市聚集。

(七）全力增进人民福祉

1.稳定和扩大就业。加强农民技能培训等就业服务，切实抓好高校毕业生、农村富余劳动力和城乡困难群体就业工作，新增就业7万人。优化全民创业环境，增加创业贷款，鼓励创业带动就业。多渠道增加农民财产性收入，加大对苇场职工等低收入群众扶贫力度。

2.完善社会保障。推进基本养老、基本医疗保险城乡并轨，完善城乡居民大病保险制度，提高社会保险、城乡低保和优抚标准。高度重视养老工作，推进机构养老、居家养老、社区养老，鼓励社会力量参与养老事业。推进残疾人康复中心加快建设。

3.改善居民居住条件。加快保障性住房建设和农垦职工危房改造，支持辽河油田边远矿区职工迁转，下气力解决回迁楼问题。改造新建水热气管网210公里。科学布局公交线路，更新公交车150台。

4.支持教育优先发展。加大基础教育投入，妥善解决小学、中学建设布局问题。推进义务教育学校标准化。推进素质教育，减轻学生课业负担。力争两年实现公办 幼儿园城乡全覆盖。深化校企合作，组建盘锦职业教育集团。健全家庭经济困难学生资助体系，让每一名学生都公平享有受教育的权利。鼓励支持华发国际学校、富 盈国际学校等民办学校发展。

5.丰富群众精神文化生活。简朴、务实、隆重地办好纪念建市30周年系列活动。办好全国十二届美展港澳台作品展。启动运行少儿科普乐园。积极开展文化惠民活动，繁荣演出市场。做好中超联赛辽足主场比赛服务，高水平承办辽宁第八届少数民族传统体育运动会。

6.提高人民群众健康水平。加强村镇和社区卫生医疗机构建设，推动优质医疗资源向基层流动。新农合筹资标准提高到400元。继续抓好出生缺陷干预工程、贫困白内障患者免费救治等医疗惠民项目。启动建设市精神病医院。建立严格的全程监管制度，确保群众饮食用药安全。

(八）加强民主法治和政府自身建设

自觉接受人大及其常委会法律监督、工作监督和政协民主监督，认真办理人大代表议案、建议和政协提案，广泛听取各民主党派、工商联、无党派人士的意见和建 议。坚持依法行政，规范行政行为。加大行政监察、审计监督、政务督查力度，推进政务公开，加强行政复议，努力建设法治型和服务型政府。

深入推进社会治理创新试点工作。加强基层社会服务管理体系建设，提升治理水平和公共服务能力。强化社会治安综合治理，依法防范和惩治违法犯罪活动。推进 “六五”普法。健全重大决策风险评估机制，完善信访和调解工作体系，高度重视安全生产、突发公共事件应急处置等工作，全力维护社会和谐稳定。

积极支持驻盘部队、国防和后备力量建设，深入开展双拥共建工作。努力做好民族宗教、防震减灾、人民防空、妇女儿童、老龄慈善、统计、物价等各项工作。

各位代表：新一届政府即将开始履行职责，我们一定肩负重任，不负人民的重托。

我们要深入开展群众路线教育实践活动，时刻以人民利益为重，雷厉风行，真抓实干，用实实在在的业绩，接受党和人民的检验。

我们要勇于担当，勇挑重担，主动走进矛盾，破解难题，把发展的事情一件一件地做好，打造敢担当、有作为的政府。

我们要开拓创新，解放思想，大胆实践，把盘锦发展大业不断推向前进。

我们要廉洁从政，带头执行中央八项规定，坚决反对“四风”，从严管控财政支出，大力压缩公用经费，厉行节约。坚持用制度管权管事管人，依法严惩腐败行为，建设廉洁政府。

各位代表，新的形势催人奋进，新的征程任重道远。让我们在市委的坚强领导下，紧紧团结依靠全市人民，戮力同心，奋勇拼搏，为创造盘锦更加幸福美好的明天而努力奋斗！
